# Associations between PIK3CA Mutations and Disease Free Survival in Patients with HR+, HER2− Tumors Treated with Adjuvant Hormonal Therapy: A Real-World Study in Croatia

**DOI:** 10.1155/2024/5648845

**Published:** 2024-09-14

**Authors:** Dora Čerina Pavlinović, Natalija Dedić Plavetić, Ingrid Belac Lovasić, Robert Šeparović, Josipa Flam, Marija Pancirov, Žarko Bajić, Snježana Tomić, Eduard Vrdoljak

**Affiliations:** ^1^ Department of Oncology University Hospital Center Split School of Medicine University of Split, Split, Croatia; ^2^ Department of Oncology University Hospital Center Zagreb School of Medicine University of Zagreb, Zagreb, Croatia; ^3^ School of Medicine University of Rijeka, Rijeka, Croatia; ^4^ Department of Medical Oncology Sestre Milosrdnice University Hospital Centre University Hospital for Tumors, Zagreb, Croatia; ^5^ Department of Radiotherapy and Oncology University Hospital Center Osijek School of Medicine University of Osijek, Osijek, Croatia; ^6^ Research Unit “Dr. Mirko Grmek” Psychiatric Clinic “Sveti Ivan”, Zagreb, Croatia; ^7^ Department of Pathology University Hospital Center Split School of Medicine University of Split, Split, Croatia

## Abstract

**Introduction:**

Disease recurrence in patients with the early hormone receptor-positive (HR+), human epidermal growth factor receptor 2-negative (HER2−) breast tumor subtype is particularly challenging to manage due to its complex and very heterogeneous biological nature. Namely, due to primary and secondary resistance, one-quarter of patients with early-stage disease will experience disease recurrence. This variability in the timing of recurrence highlights the need to better identify key biomarkers that could predict therapeutic outcomes and guide personalized treatment strategies for these patients. Mutations in the phosphatidylinositol 4,5-bisphosphate 3-kinase catalytic subunit alpha (PIK3CA) gene are highly prevalent (30–40%) in HR+/HER2− advanced breast cancer. They lead to activation of the PI3K/AKT/mTOR pathway, promoting cell growth, and proliferation, and are associated with poor prognosis in advanced breast cancer. Our aim was to examine the association between and impact of PIK3CA mutation status on disease-free survival (DFS) in HR+/HER2− early breast cancer patients.

**Methods:**

This cohort study was multicentric and retrospective in nature and was conducted at five Croatian institutions from July 2020 to December 2021. The study included initially early and locally advanced operable HR+/HER2− breast cancer patients who were diagnosed with disease recurrence during adjuvant hormonal treatment or within the first six years of follow-up.

**Results:**

A total of 186 patients were included, 40.9% of whom tested positive for the PIK3CA mutation. Primary and adjuvant treatment, particularly adjuvant endocrine treatment, were similar between the two groups. After adjustment for 14 relevant covariates, we found that patients with a positive PIK3CA status and the H1047 PIK3CA mutation had a significantly lower hazard of disease recurrence than patients with no PIK3CA mutation (HR 0.65; 95% CI 0.45; 0.95; *p*=0.024; false discovery rate, FDR <10%).

**Conclusions:**

This study highlights the potential impact of PIK3CA mutations on disease recurrence during or following adjuvant endocrine therapy and potentially opens the door for further investigation of possibly more personalized treatment strategies.

## 1. Introduction

Breast cancer accounts for almost 12% of all cancers diagnosed in women and is the leading cause of female cancer-related death worldwide [1, 2]. Unfortunately, despite recent significant improvements in outcomes due to modern adjuvant therapies, approximately 25% of women with early hormone receptor-positive (HR+), human epidermal growth factor receptor 2-negative (HER2−) tumor experience disease recurrence [3, 4]. Advanced stages of this immunohistochemical subtype are particularly challenging to manage due to its complex and very heterogeneous biological nature [4]. Adjuvant endocrine treatment plays a pivotal role in the management of HR+/HER2− early breast cancer with the aim of suppressing the proliferation of hormone-dependent tumor cells in order to prevent or delay disease progression [5]. There is considerable variability in the timing of disease progression relative to the initiation of adjuvant endocrine therapy. Primary endocrine resistance is characterized by recurrence during the first two years of adjuvant endocrine therapy, while secondary resistance implies recurrence after more than two years since the initiation of endocrine therapy [6]. This variability in timing highlights the need to better identify key biomarkers that could predict therapeutic outcomes and guide new, more personalized adjuvant treatment strategies [7]. Currently, research on this topic is expanding every day and existing investigations utilize many techniques beyond immunohistochemical analysis of early breast cancer, such as gene assays and immune marker analysis to predict treatment response and long-term outcomes [8–10]. Furthermore, recent findings have shown the benefit of targeted therapy as an adjuvant treatment in patients with a higher risk (HR+/HER2−) [11, 12]. Mutations in the phosphatidylinositol 4,5-bisphosphate 3-kinase catalytic subunit alpha (PIK3CA) gene are highly prevalent (30–40%) in patients with HR+/HER2− advanced breast cancer [13–15]. They lead to activation of the PI3K/AKT/mTOR pathway, promoting cell growth, and proliferation, and are associated with poor prognosis in advanced breast cancer [16]. Moreover, there is emerging evidence that PIK3CA mutations may influence the response to endocrine therapy [17]. Fortunately, targeted therapy with PIK3CA inhibitors has been shown to clinically benefit in patients with metastatic PIK3CA mutated breast cancer [18]. However, the association between PIK3CA mutations and their impact on the timing of disease recurrence in relation to adjuvant endocrine treatment remains undefined and requires further investigation [19, 20]. Given that the importance of real-world outcomes and results are more acknowledged than ever before, we investigated the association between PIK3CA mutations and the time of disease recurrence in relation to adjuvant endocrine treatment in HR+/HER2− negative early and locally advanced breast cancer patients using data from everyday clinical practice in Croatia.

## 2. Methods

### 2.1. Study Design

We performed this analysis on a subset of data from a larger, multicenter, retrospective cohort of breast cancers tested for PIK3CA mutations as a part of the testing for eligibility for a treatment with the PIK3CA inhibitor Alpelisib. Nevertheless, the patients included in this analysis were not part of any clinical trial nor they have received the treatment with Alpelisib during the investigated period. This study was multicentric and retrospective in nature and was conducted from July 1, 2020 to December 31, 2021, at five Croatian university hospitals in Split, Zagreb, Osijek, and Rijeka. The study was approved by the ethics committees, and it was in accordance with the World Medical Association's Declaration of Helsinki of 1975 as revised in 2013 [21]. The study protocol was not preregistered.

### 2.2. Study Population

The target population was women diagnosed with HR+/HER2− early or locally advanced breast cancer who had experienced disease recurrence during or after endocrine-based adjuvant treatment. Therefore, patients had to have early-stage or locally advanced operable breast cancer at the time of initial diagnosis but also had to have subsequent disease recurrence, i.e., evidence of endocrine resistance. Thus, the exclusion criteria were metastatic disease at initial diagnosis and no recurrence during the first six years of follow-up. We did not select the sample but evaluated the entire eligible population that was tested at five study centers during the enrollment period. We did not perform a power analysis before the data were collected.

### 2.3. Endpoints

The primary endpoint was disease free survival (DFS) from the initiation of adjuvant endocrine therapy to the time of disease recurrence or death from any cause. The secondary outcomes were late recurrence, defined as recurrence or death from any cause more than one year after completion of five years of adjuvant endocrine therapy, and overall survival (OS) from the initiation of adjuvant endocrine therapy to death from any cause. Both DFS and OS are presented in years.

### 2.4. Exposure

Primary exposure was PIK3CA mutation status, operationalized as a binary variable. The secondary exposure was specific to the PIK3CA mutation. Due to the insufficient sample size and specific PIK3CA mutation prevalence, we grouped the N345, C420, E546, and G1049 mutations into one variable named “other PIK3CA mutations.” We obtained DNA from formalin-fixed, paraffin-embedded (FFPE) tumor samples. Tissue selection was based on archival availability and quality and included both primary tumors and metastatic sites. In three patients, we obtained circulating cell-free DNA from blood plasma. Oncologists identified and referred eligible patients for testing. We extracted DNA from FFPE samples using the Cobas® DNA Sample Preparation Kit. Only sections with a tumor content of 10% or more were considered suitable. For samples with lower tumor content, microdissection was used to increase the tumor content in the final section to a minimum of 30%. The extraction process included deparaffinization, tissue lysis, protein digestion, and DNA purification. The resulting concentration of genomic DNA was standardized to 2 ng/*μ*l. For cell-free DNA (cfDNA) extraction, plasma was separated from whole blood by centrifugation. The Roche High Pure PCR Template Preparation Kit facilitated the extraction using a protocol similar to that described above. The final DNA concentration was adjusted to 2 ng/*μ*l. The Cobas® PIK3CA Mutation Testing Kit was used for real-time PCR-based amplification and mutation detection on the Cobas z 480 analyzer. This kit provides high analytical sensitivity for FFPE tissue, detecting mutations with 95–100% accuracy at a minimum of 5% mutations in a 50 ng DNA sample. The kit contains primers targeting specific sequences in the PIK3CA exons and an internal control. Each run included a PIK3CA mutant control and a negative control. The detection mechanism is based on fluorescence measurements after dye quencher separation. The final readout indicates the codon position without detailing the specific base substitution.

The HR status (estrogen and progesterone) and HER2 status were determined using immunohistochemistry in accordance with the current American Society of Clinical Oncology/College of American Pathologists (ASCO/CAP) guidelines in all five laboratories where PIK3CA analysis was performed. The analysis was not centralized because all laboratories have equal internal and external quality assessment tests performed regularly and they all receive a NORDIQ certificate once a year.

### 2.5. Covariates

We adjusted the analysis for study center, age, pathohistological tumor type, tumor size, number of affected lymph nodes, tumor grade, estrogen and progesterone receptor status, neoadjuvant treatment, primary surgery, adjuvant radiotherapy, adjuvant chemotherapy, treatment with bisphosphonates, and specific adjuvant endocrine therapy.

### 2.6. Statistical Analysis

We analyzed both differences in DFS between patients with negative and positive PIK3CA status and between patients with specific PIK3CA mutations using Cox, proportional hazard regression, as well as differences in the late recurrences using binary logistic regression. In all cases, the referent category was negative PIK3CA status. First, we performed a bivariate analysis followed by a multivariable analysis adjusted for study center, age, pathohistological tumor type, tumor size, number of affected lymph nodes, grade, estrogen and progesterone receptor status, neoadjuvant treatment, primary surgery, adjuvant radiotherapy, adjuvant chemotherapy, treatment with bisphosphonates, and specific adjuvant endocrine therapy. Finally, we performed multivariable analysis with adjustment for the same variables but after multiple imputation of missing data. In bivariate analysis, we presented the median DFS/OS with 95% confidence intervals (CI) or number and percentage of participants who have experienced the late recurrence. As results of Cox regression, we presented hazard ratios (HR) with their 95% CIs, and as a result of logistic regression, we presented odds ratios (OR) with their 95% CIs. We tested the hypothesis that the missing data were missing completely at random (MCAR) using Little's test and later assumed that the data were missing at random (MAR) with an ignorable missing data mechanism. We conducted multiple imputations using sequential regression multivariate imputation or chained equations (MICE) with the sequence of imputation models for all variables and used as predictors for each imputed variable. Imputations were conducted in order from the variable with the least missing data to the one with the most. As predictors with no missing data, we included PIK3CA status, study center, tumor stage at diagnosis, number of affected lymph nodes, neoadjuvant treatment, and treatment with surgery, radiation therapy, chemotherapy, and specific adjuvant endocrine therapy. To enable reproduction of the results, we set a random-number seed at 4112000. We controlled for false-positive rates using the Benjamini–Hochberg procedure, with a preset false discovery rate (FDR) of <10%. We set two-tailed statistical significance at *p* < 0.05 and calculated all CIs at the 95% level. Statistical data analysis was performed using StataCorp 2019 (Stata Statistical Software: Release 16.1. College Station, TX: StataCorp LLC).

## 3. Results

### 3.1. Sample Characteristics

A total of 186 women were included in the study; 76 (40.9%) were positive for the PIK3CA mutation and 110 (59.1%) were negative (Table 1). H1047 and E545 were the two most prevalent hotspot mutations and were found in 19.4% and 15.1% patients, respectively. The median age was 64 years (IQR 58–71 years) in the PIK3CA-positive group and 63 years (IQR 54–72 years) in the PIK3CA-negative group. Patients with PIK3CA mutations had somewhat more prevalent ductal tumors of lower grades that were more often progesterone positive. Primary and adjuvant treatment, particularly adjuvant endocrine treatment, were similar between the two groups (Table 1). The toxicity and withdrawal of patient consent as a reason for discontinuation of adjuvant endocrine therapy were also comparable between the PIK3CA-positive and PIK3CA-negative groups. Information on PIK3CA status was collected for all patients.

Data on at least one covariate for which we planned to adjust the analyses were missing in 22 patients (11.8%). The proportion of missing data varied between study centers. Patients with missing data were somewhat older, more often diagnosed with ductal tumor type, IIIA or IIIB stage at diagnosis, somewhat larger tumors, and had more affected lymph nodes. Patients with at least one missing data point on planned covariates were more often treated with tamoxifen-only than with aromatase inhibitors-only and had a longer duration of follow-up. For these reasons, it was necessary to repeat the main, adjusted analysis with multiple imputation of missing data.

### 3.2. Disease-free Survival

**Figure 1:**
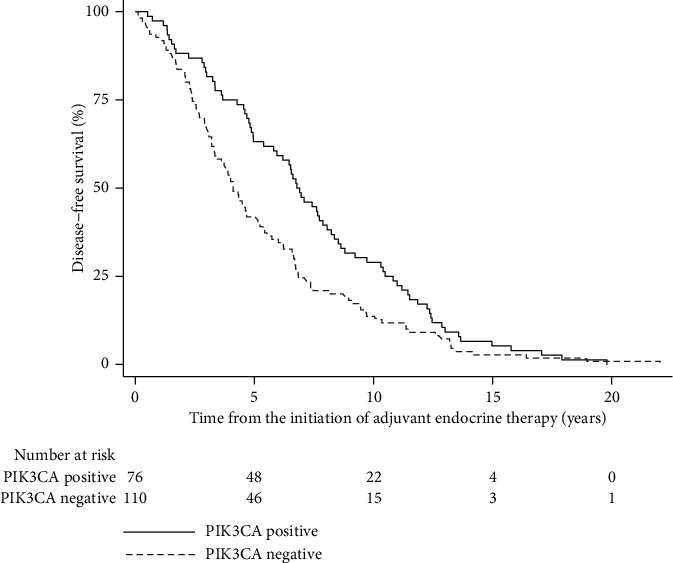
Kaplan–Meier curves of disease-free survival from the initiation of adjuvant endocrine therapy (years) by PIK3CA status (*n* = 186).

**Figure 2:**
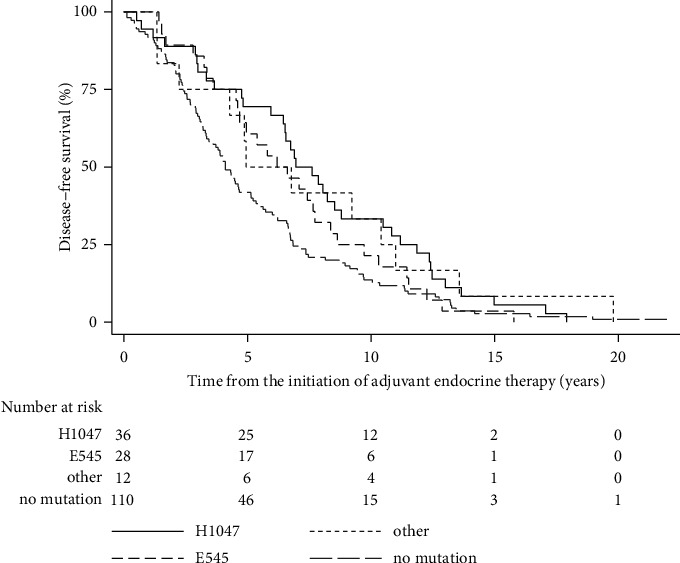
Kaplan–Meier curves of disease-free survival from the initiation of adjuvant endocrine therapy (years) by hotspot mutation (*n* = 186).

The median DFS was statistically significantly longer in the PIK3CA mutation group (6.8 years [95% CI 5.4; 8.1]) than in the nonmutation group (4.1 years [95% CI 3.3; 5.1]) (Table 2, Figure 1). The hazard for disease recurrence in the group with PIK3CA mutations was 0.67 (95% CI 0.50; 0.90) compared to the hazard in the group without PIK3CA mutations (*p*=0.008; FDR <10%). After adjustment for the aforementioned covariates, and after multiple imputation of missing covariate data, the hazard ratio for recurrence remained statistically significantly different between the two examined groups, but in the analysis with imputed missing data, the FDR was >10% (Table 2). Patients with a specific PIK3CA mutation, H1047, had a statistically significantly lower risk of recurrence than patients without a PIK3CA mutation according to both the bivariate analysis and multivariate analysis after adjustment for the mentioned covariates and after multiple imputation of missing data (Table 3, Figure 2).

### 3.3. Late Recurrence

According to bivariate, unadjusted analysis, late recurrence was observed in 45 (59.2%) of PIK3CA-positive patients compared to 39 (35.5%) of PIK3CA-negative patients, resulting in an unadjusted OR of 2.64 (95% CI 1.45; 4.82; *p*=0.002; FDR <10%) (Table 2). After adjustment for the mentioned covariates and after multiple imputation of missing data, the OR was no longer statistically significant. Patients with H1047 PIK3CA mutations had statistically significantly higher odds of late recurrence than patients without PIK3CA mutations according to bivariable and multivariable adjusted analyses and analysis with multiple imputation of missing covariate data; however, in the last analysis, the FDR was >10% (Table 3).

### 3.4. Overall Survival

The median survival was not reached, and no difference in overall survival between the studied groups was found to be statistically significant.

## 4. Discussion

The results of our study contribute to the growing body of evidence regarding the role of PIK3CA mutations in early breast cancer prognosis and treatment response. Our analysis revealed that 40.9% of patients were positive for the PIK3CA mutation, which is consistent with the findings of previous studies indicating a high prevalence of these mutations in HR+/HER2− advanced breast cancer [13–15]. Notably, the H1047R mutation was the most common mutation in PIK3CA-positive patients, followed by the E545K mutation which is also in line with the findings of previous studies [15, 22].

The main finding of our study was the association between H1047R PIK3CA mutation and DFS and late recurrence. In other words, the presence of this specific mutation was associated with better treatment outcomes and a lower risk of disease recurrence. This potentially indicates that this specific mutation could be defined as positive prognostic factor in HR+/HER2− early breast cancer. However, the direction of this association is opposite to the conclusions of a 2018 meta-analysis by Sobhani et al., which revealed a negative prognostic effect of PIK3CA mutations [16]. This inconsistency probably indicates differences in the prognostic and predictive validity or direction of effect of the PIK3CA mutations on different pathohistological subtypes of breast cancer, at different stages, and in relation to different therapies. The conclusion of Sobhani et al. was based on a pooled sample with high heterogeneity. Our main findings contradict the results of another large meta-analysis by Fillbrunn et al. which was performed on more homogeneous samples than that of Sobhani et al. and which also revealed a negative prognostic and predictive effect of PIK3CA mutation in advanced HR+/HER2− breast cancer [23]. However, our results are more in line with the findings of a pooled analysis of data from 19 studies on 10319 individual patients with early breast cancer which found the beneficial, positive effect of PIK3CA mutation on invasive disease-free survival, as well as with the findings of several smaller primary studies [24, 25]. The association between particularly H1047R PIK3CA mutation and better outcomes is indeed intriguing, and there could be several factors, including less aggressive phenotypes, lower histopathological tumor grade, and sensitivity to hormonal or targeted therapy, as well as presence of other mutations affecting the signaling pathway [26, 27]. Furthermore, some of the less prevalent mutations such as C420R are related to better prognosis than other PIK3CA mutations but without difference in comparison to PIK3CA wild-type status [28]. Apart from these conflicting results, several studies have shown no prognostic value of PIK3CA mutation [29].

Although targeted therapy is already becoming the optimal treatment for the early breast cancer, PIK3CA inhibitors remain the gold standard for treating metastatic disease [11, 12, 18]. While some studies on PIK3 inhibitors have shown that they are beneficial in the neoadjuvant setting, their applicability in the early phases remains unclear [30]. Our study highlights the potential importance of determining PIK3CA mutational status in early breast cancer, especially in research stetting, and by that indicates the need for further research regarding the use of PIK3CA inhibitors in the neo/adjuvant treatment strategy.

Finally, the H1047R mutation was significantly associated with late recurrence, highlighting its potential role in the course of disease. The real-world setting of our study provides a unique perspective that reflects the diverse patient characteristics and treatment approaches used in everyday clinical practice. While controlled clinical trials provide valuable insights, they often have strict inclusion criteria that may not represent the full spectrum of patients seen in routine care. Our findings are derived from a real-world cohort and may therefore provide more generalizable insights for clinicians and researchers.

### 4.1. Study Limitations

Our study has several limitations. The retrospective study design introduced the relative imprecision in our endpoints because we had to use routinely collected data. In addition, although we adjusted for several confounding variables in our multivariable analysis, the possibility of residual confounding could not be excluded such as the Ki-67 index of cellular proliferation which could be related to PIK3CA status and could possibly affect its prognostic value. That is why, future prospective studies with larger sample sizes and diverse populations are needed to validate our findings and address these potential confounding factors.

## 5. Conclusion

In conclusion, our study highlights the potential prognostic value of PIK3CA mutations, particularly H1047R, for predicting disease recurrence among women with HR+/HER2- early or locally advanced operable breast cancer. These findings could pave the way for further research into the clinical implications of PIK3CA mutations and their potential as prognostic or therapeutic targets in early breast cancer care.

## Figures and Tables

**Table 1:** Sample characteristics according to PIK3CA mutation status (*n* = 186).

	PIK3CA status	
	Positive (*n* = 76)	Negative (*n* = 110)
Age (years), median (IQR)	64 (58; 71)	63 (54; 72)
Pathohistological tumor type		
Ductal	45 (59.2)	40 (36.7)
Lobular	13 (17.1)	17 (15.6)
NOS	17 (22.4)	46 (42.2)
Special types^∗^	1 (1.3)	6 (5.5)
PIK3CA mutation		
H1047	36 (47.4)	0 (0.0)
E545	28 (36.8)	0 (0.0)
Other^†^	12 (15.8)	0 (0.0)
No PIK3CA mutation	0 (0.0)	110 (100.0)
Tissue samples		
Archive tissue	64 (84.2)	77 (70.0)
Last available biopsy	11 (14.5)	33 (30.0)
Blood	1 (1.3)	0 (0.0)
Stage at diagnosis		
IA	18 (23.7)	26 (23.6)
IB	7 (9.2)	12 (10.9)
IIA	16 (21.1)	16 (14.5)
IIB	15 (19.7)	16 (14.5)
IIIA	11 (14.5)	17 (15.5)
IIIB	3 (3.9)	11 (10.0)
IIIC	6 (7.9)	12 (10.9)
Tumor size (cm), median (IQR)	2.4 (1.8; 4.0)	2.5 (1.5; 3.5)
Number of affected lymph nodes		
0	24 (31.6)	35 (31.8)
1	29 (38.2)	35 (31.8)
≥2	23 (30.3)	40 (36.4)
Grade		
1	12 (16.4)	5 (4.8)
2	47 (64.4)	70 (66.7)
3	14 (19.2)	30 (28.6)
Estrogen positive (%), median (IQR)	90 (70; 100)	95 (80; 100)
Progesterone positive (%), median (IQR)	70 (15; 90)	48 (6; 90)
Neoadjuvant treatment	8 (10.5)	16 (14.5)
Primary surgery	73 (96.1)	104 (94.5)
Radiotherapy	64 (84.2)	94 (85.5)
Adjuvant chemotherapy	59 (77.6)	84 (76.4)
Bisphosphonates	6 (8.1)	9 (8.2)
AET		
Tamoxifen	14 (18.7)	31 (28.2)
AI	39 (52.0)	57 (51.8)
Tamoxifen and AI	22 (29.3)	22 (20.0)
Duration of AET (years), median (IQR)	5.0 (3.6; 5.6)	4.0 (2.3; 5.1)
Reason for AET discontinuation		
Completed 5-years treatment	34 (45.3)	30 (27.8)
Progression	36 (48.0)	68 (63.0)
Toxicity	1 (1.3)	1 (0.9)
Patient withdrawal	4 (5.3)	9 (8.3)
Death	15 (19.7)	17 (15.5)
Duration of follow-up (years), median (IQR)	12 (7.15)	8 (5.12)

The data are presented as the number (percentage) of patients unless otherwise stated. UHC, University Hospital Center; IQR, interquartile range; NOS, not otherwise specified; AI, aromatase inhibitor; AET, adjuvant endocrine therapy. ^∗^Special types were micropapillary (*n* = 3), mixed (*n* = 2), and others (*n* = 2). ^†^Others were E542 (*n* = 5), N345 (*n* = 6), and G1049 (*n* = 1).

**Table 2:** Study outcomes by PIK3CA status (*n* = 186).

	PIK3CA status				*p*
	Positive (*n* = 76)		Negative (*n* = 110)		
Primary outcome					
DFS (years), median (95% CI)	6.8	(5.4; 8.1)	4.1	(3.3; 5.1)	
Unadjusted HR (95% CI)	0.67	(0.50; 0.90)	1.00	Referent	0.008^∗^
Adjusted HR (95% CI)^†^	0.65	(0.45; 0.95)	1.00	Referent	0.024^∗^
Imputed, adjusted HR (95% CI)^‡^	0.68	(0.48; 0.99)	1.00	Referent	0.045
Secondary outcomes					
Late recurrence, *n* (%)	45	(59.2)	39	(35.5)	
Unadjusted OR (95% CI)	2.64	(1.45; 4.82)	1.00	Referent	0.002^∗^
Adjusted OR (95% CI)^†^	2.27	(0.97; 5.35)	1.00	Referent	0.060
Imputed, adjusted HR (95% CI)^‡^	2.64	(1.45; 4.82)	1.00	Referent	0.083
OS (years), median (95% CI)	21.1	(16.6; n.r.)	n.r.	(14.6; nr.)	
Unadjusted HR (95% CI); *p*	0.88	(0.44; 1.76)	1.00	Referent	0.713
Adjusted HR (95% CI); *p*^†^	0.62	(0.20; 1.90)	1.00	Referent	0.401
Imputed, adjusted HR (95% CI)^‡^	1.21	(0.42; 3.44)	1.00	Referent	0.724

DFS, disease-free survival; CI, confidence interval; HR, hazard ratio calculated using Cox proportional hazard regression; OR, odds ratio calculated via binary logistic regression; n.r., not reached. ^∗^False discovery rate <5%. ^†^Adjusted for study center, age, pathohistological tumor type, tumor size, number of affected lymph nodes, grade, estrogen and progesterone receptor status, neoadjuvant treatment, primary surgery, radiotherapy, adjuvant chemotherapy, treatment with bisphosphonates, and specific adjuvant endocrine therapy; *n* = 164 because of missing data. ^‡^Analysis was carried out on the multiply imputed data and adjusted for the mentioned covariates, *n* = 186.

**Table 3:** Study outcomes according to specific PIK3CA mutation status (*n* = 186).

	PIK3CA mutation									PIK3CA negative (*n* = 110)	
	H1047 (*n* = 36)			E545 (*n* = 28)			Other (*n* = 12)				
Primary outcome											
DFS (years), median (95% CI)	7.0	(5.9; 8.8)		6.2	(4.6; 7.7)		4.9	(1.3; 11.0)		4.1	(3.3; 5.1)
Unadjusted HR (95% CI); p	0.62	(0.42; 0.91)	0.014^∗^	0.77	(0.51; 1.18)	0.230	0.62	(0.34; 1.14)	0.123	1.00	Referent
Adjusted HR (95% CI); *p*^†^	0.47	(0.29; 0.77)	0.002^∗^	0.89	(0.54; 1.47)	0.656	0.97	(0.43; 2.21)	0.942	1.00	Referent
Imputed, adjusted HR (95% CI)^‡^	0.50	(0.31; 0.80)	0.004^∗^	0.88	(0.53; 1.47)	0.634	1.11	(0.54; 2.28)	0.778	1.00	Referent
Secondary outcomes											
Late recurrence, *n* (%)	24	(66.7)		15	(53.6)		6	(50.0)		39	(35.5)
Unadjusted OR (95% CI); *p*	3.64	(1.64; 8.07)	0.001^∗^	2.10	(0.91; 4.86)	0.083	1.82	(0.55; 6.03)	0.327	1.00	Referent
Adjusted OR (95% CI); *p*^†^	3.72	(1.15; 12.01)	0.028^∗^	1.78	(0.49; 6.40)	0.378	0.88	(0.13; 5.87)	0.898	1.00	Referent
Imputed, adjusted HR (95% CI)^‡^	3.48	(1.14; 10.61)	0.029	1.53	(0.48; 4.84)	0.472	0.81	(0.13; 5.06)	0.825	1.00	Referent
OS (years), median (95% CI)	n.r.	(14.7; n.r.)		21.1	(21.1; n.r.)		15.4	(6.1; n.r.)		n.r.	(14.5; n.r.)
Unadjusted HR (95% CI); *p*	0.56	(0.21; 1.52)	0.255	0.98	(0.36; 2.65)	0.963	1.64	(0.60; 4.48)	0.331	1.00	Referent
Adjusted HR (95% CI); *p*^†^	0.16	(0.03; 0.81)	0.027^∗^	3.06	(0.72; 13.01)	0.129	1.36	(0.16; 11.86)	0.779	1.00	Referent
Imputed, adjusted HR (95% CI)^‡^	0.29	(0.06; 1.53)	0.145	5.80	(1.43; 23.61)	0.014	2.79	(0.48; 16.06)	0.252	1.00	Referent

DFS, disease-free survival; CI, confidence interval; HR, hazard ratio calculated using Cox proportional hazard regression; MI, multiple imputation; OR, odds ratio calculated via binary logistic regression; OS, overall survival; n.r., not reached. ^∗^False discovery rate <5%. ^†^Adjusted for study center, age, pathohistological tumor type, tumor size, number of affected lymph nodes, grade, estrogen and progesterone receptor status, neoadjuvant treatment, primary surgery, radiotherapy, adjuvant chemotherapy, treatment with bisphosphonates, and specific adjuvant endocrine therapy; *n* = 164 because of missing data. ^‡^Analysis was carried out on the multiply imputed data and adjusted for the mentioned covariates, *n* = 186.

## Data Availability

The data and Stata code are available from the corresponding author upon request.
